# Genetic and Antigenic Diversity of *Bubaline alphaherpesvirus 1*

**DOI:** 10.3390/v17081110

**Published:** 2025-08-13

**Authors:** Rocío Lucía Tau, Ana Eugenia Marandino, Fátima Torales, Fabrício Souza Campos, Paulo Michel Roehe, José Luis Konrad, Sonia Alejandra Romera, Ruben Pérez, Silvina Soledad Maidana

**Affiliations:** 1 Institute of Virology and Technological Innovations, Dr. Nicolas Repetto and De los Reseros, IVIT (INTA-CONICET), Hurlingham 1686, Argentina; torales.fatima@inta.gob.ar (F.T.); romera.alejandra@inta.gob.ar (S.A.R.); 2 Evolutionary Genetics Section, Faculty of Sciences, Institute of Biology, University of the Republic, Montevideo 11400, Uruguay; amarandino@fcien.edu.uy (A.E.M.); rperez@fcien.edu.uy (R.P.); 3 Laboratory of Bioinformatics & Biotechnology, Department of Microbiology, Immunology and Parasitology, Institute of Basic Health Sciences (ICBS), Universidade Federal do Rio Grande do Sul (UFRGS), Rio Grande do Sul 90050-170, Brazil; camposvet@gmail.com; 4 Laboratory of Virology, Department of Microbiology, Immunology and Parasitology, Institute of Basic Health Sciences (ICBS), Universidade Federal do Rio Grande do Sul (UFRGS), Rio Grande do Sul 90050-170, Brazil; proehe@gmail.com; 5 Department of Animal Production, Faculty of Veterinary Sciences, Northeast National University (UNNE), Corrientes 3400, Argentina; joseluis.konrad.vet@comunidad.unne.edu.ar; 6 Faculty of Agricultural and Veterinary Sciences, Veterinary Research Institute, University of the Salvador, Buenos Aires 1426, Argentina

**Keywords:** *Bubaline alphaherpesvirus 1*, genetic diversity, antigenic variation, cross-neutralization, phylogenetic analysis, recombination, water buffalo

## Abstract

*Bubaline alphaherpesvirus 1* (BuHV-1) is a virus that belongs to the *Varicellovirus* genus within the *Alphaherpesvirinae* subfamily. While BuHV-1 infections in water buffaloes (*Bubalus bubalis*) are often subclinical, clinical manifestations have been reported. This study provides complete genome sequences of five BuHV-1 strains isolated in Argentina, marking the first genomic characterization of BuHV-1 from the Americas. Phylogenetic reconstructions based on whole-genome and coding sequences, along with analyses of glycoproteins C, D, and E, identified a distinct clade and divergent strains. Comparative genomic analyses with publicly available BuHV-1 and *Bovine alphaherpesvirus 5* (BoHV-5) sequences showed nucleotide divergence of up to 1.3% among BuHV-1 strains, indicating significant intraspecific genetic diversity. Cross-neutralization assays revealed variable relationships between BuHV-1 and BoHV-5 strains. Some Argentinian BuHV-1 strains exhibited significant antigenic subtype differences compared to *Bovine alphaherpesvirus 1* (BoHV-1). Recombination analyses uncovered events between BuHV-1 and bovine herpesviruses, suggesting a complex evolutionary history within mixed farming systems. The findings indicate that the monophyletic BuHV-1 clade, including the reference BuHV-1 isolate, is representative of the BuHV-1 species. The remaining strains, provisionally classified as BuHV-1 indeterminate (BuHV-1i), can be categorized based on specific clinical and antigenic properties. The identified heterogeneity has significant implications for diagnostic accuracy, vaccine development, and disease management strategies in buffalo populations worldwide.

## 1. Introduction

Water buffaloes (*Bubalus bubalis*) are a vital livestock species, contributing to global agricultural economies through milk production, meat supply, and draft power. With a worldwide population exceeding 230 million across 67 countries, buffaloes are particularly remarkably esteemed in tropical and subtropical regions for their adaptability to extreme climatic conditions, natural disease resistance, and ability to utilize low-quality fodder [1,2]. In Asia, countries such as India and China lead in buffalo production, while in South America, nations like Brazil and Argentina are experiencing rapid growth in buffalo farming, driven by increasing demand for buffalo-derived products. The global buffalo industry faces challenges from infectious diseases, which can affect productivity and economic sustainability. Understanding pathogen dynamics within buffalo populations is therefore crucial, especially within the One Health framework that emphasizes the interconnected nature of human, animal, and environmental health systems [3]. Among the viral pathogens impacting buffaloes, herpesviruses are a significant concern due to their ability to establish lifelong latent infections and their potential for cross-species transmission [4].

*Bubaline alphaherpesvirus 1* (BuHV-1) is a double-stranded DNA virus and is classified within the *Alphaherpesvirinae* subfamily and the *Varicellovirus* genus. First isolated in 1972 from an asymptomatic buffalo in Australia (B6 strain), additional strains of BuHV-1 were later identified following corticosteroid-induced reactivation in 2004 [5,6]. Subsequent reports have detected BuHV-1 in various clinical contexts, including mild respiratory signs, vulvovaginitis, and aborted fetuses, although subclinical infections are more common [7,8,9,10]. The virus has been reported in countries such as Brazil, Iran, India, Italy, Australia, and Argentina, indicating a wide geographical distribution [10,11,12,13]. Despite its prevalence, the epidemiological and pathogenic roles of BuHV-1 remain underexplored, highlighting the need for comprehensive genetic and antigenic studies.

The BuHV-1 genome spans approximately 137 kb, with guanine–cytosine (G+C) content of 76.8%, and encodes 70 putative genes, including duplicated copies of BICP4 and BICP22 [6]. Phylogenetically, BuHV-1 demonstrates close relationships with bovine alphaherpesviruses, sharing 88.9% nucleotide identity with *bovine herpesvirus 1* (BoHV-1) and 95.9% identity with *bovine herpesvirus 5* (BoHV-5) [14]. These substantial sequence similarities create significant challenges for diagnostic differentiation, as cross-neutralization between BuHV-1 and BoHV-1 can lead to false-positive results in diagnostic assays, potentially compromising control programs designed for bovine herpesviruses [14]. The economic impact of BuHV-1 is particularly notable in regions where buffalo farming plays a significant economic role, such as southern Italy [8,9]. Studies on virus transmission between species reveal potential for cross-species infection. For example, BuHV-1 can infect cattle, while BoHV-1 and BoHV-5 can infect buffalo. This raises significant concerns about the dynamics of viral exchange in mixed livestock production systems [13,14,15,16]. Furthermore, it has been shown that ruminant alphaherpesviruses can recombine naturally [17]. Paredes-Galarza, et al. recently suggested that BoHV-5 may have originated from recombination between bovine and buffalo herpesviruses, with water buffaloes potentially providing the conditions necessary for generating recombinant strains [18].

In Argentina, BuHV-1 was first isolated in 2014 from clinically healthy buffaloes, marking the initial detection of this virus in the Americas [12]. The Argentine buffalo population, currently nearing 150,000 animals, has experienced remarkable growth of 63% between 2013 and 2020, with population projections anticipating an increase to 432,262 animals by 2030 [16]. Located in the northeastern wetland regions, buffalo farming in Argentina complements traditional cattle production and supports integrated mixed farming systems that increase meat production resilience. Seroprevalence and molecular studies have shown a 33% prevalence of BuHV-1 in Argentine buffaloes, along with the natural co-circulation of other ruminant herpesviruses, including BoHV-1, BoHV-5, and caprine alphaherpesvirus 1 (CpHV-1), in their respective hosts, cattle and goats [19,20]. Notably, experimental studies have demonstrated that goats are susceptible to BuHV-1 infection, thereby further supporting the potential for interspecies transmission in diverse mixed farming environments [21].

The main goal of this study was to thoroughly characterize genetic diversity by comparing these isolates with available global sequences, aiming to deepen understanding of the broader evolutionary context. The study also sought to assess the antigenic diversity of BuHV-1 strains isolated in Argentina, the Australian reference strain BuHV-1 B6, and representatives of BoHV-1 and BoHV-5. By incorporating whole-genome sequencing approaches, detailed phylogenetic analyses, recombination detection methods, and comprehensive antigenic profiling through cross-neutralization assays, we provide a comprehensive assessment of BuHV-1 diversity with direct implications for diagnostic accuracy, vaccine efficacy evaluation, and disease control strategies in buffalo populations globally.

## 2. Materials and Methods

### 2.1. Sample Collection and Genome Sequencing

Five BuHV-1 strains were isolated from vaginal swabs (A549V, A067V, 84250V, PC446V) and a nasal swab (20287N) of water buffaloes in Argentina, following previously reported procedures [12]. Viral DNA was extracted using a QIAamp DNA Mini Kit (Qiagen, Hilden, Germany) and sequenced on an Illumina MiniSeq platform (Illumina, San Diego, CA, USA) at the Facultad de Ciencias, University of the Republic, Uruguay [17]. Libraries were prepared with the Nextera XT DNA Library Preparation Kit, and sequencing produced 150 bp paired-end reads. Quality control was conducted using FastQC V0.12.1, and adapters were trimmed with Trimmomatic v0.39. Reads were mapped to the BuHV-1 B6 reference genome (NC_043054.1) and BoHV-5 A663 (MW829288.1) using BWA-MEM. Consensus genomes were generated using SAMtools [22] and annotated with Prokka v1.14.6 [23], followed by manual curation.

### 2.2. Phylogenetic Analysis, Genomic Distance, and Recombination Detection

Whole-genome and CDS alignments were performed using MAFFT v7.487 with the G-INS-i algorithm [24]. Recombination events were detected using RDP4 v4.101 [25], employing seven detection methods with Bonferroni correction, and validated with SimPlot v3.5.1 [26]. Genomic distances were calculated using the Maximum Composite Likelihood model (ML) in MEGA11 [27] and kernel density plots were generated with ggplot2 in R [28,29]. Maximum likelihood phylogenetic trees were constructed using IQ-TREE v2.1.2 [30] with 10,000 ultrafast bootstrap replicates and best-fit model selection [31]. The trees were visualized using ggtree v3.0.4 [32]. Additional ML trees were generated using partial glycoprotein C (gC), D (gD), and E (gE) sequences available in GenBank, originally submitted as part of clinical case reports (see Supplementary Table S1).

### 2.3. Preparation of Hyperimmune Sera

Hyperimmune sera were produced in guinea pigs (Cavia porcellus, weighing 350–400 g) against the following eight strains: BoHV-1 (LA), BoHV-5 (2010), and BuHV-1 (B6, A549V, A067V, 84250V, PC446V, and 20287N). The viruses were obtained from clarified cell culture supernatants without additional purification. The culture supernatants were titrated and found to contain between 10^6^ and 10^8^ TCID_50_/mL of antigenic mass. They were then inactivated with 1% (*v*/*v*) 0.1 M binary bromoethylamine (BEI) at 37 °C for 25 h, after which they were emulsified at a 1:1 ratio with the adjuvant Montanide ISA 70M VG (Biogenesis Bagó, Garín, Argentina) [33]. Eight groups of five guinea pigs were housed at 25 °C with ad libitum access to food and water. Each animal received a 600 µL subcutaneous inoculation on day 15, followed by boosters on days 30 and 45. Blood was collected on days 15, 30, and 45 via saphenous vein puncture for BoHV-1 ELISA testing [34]. On day 60, terminal blood samples were obtained via cardiac puncture under anesthesia (Ketamine 50 mg/kg, Xylazine 5 mg/kg), followed by euthanasia with Pentobarbital (100 mg/kg). Euthanasia followed the final blood collection, following the approved protocol (INTA CICUAE, 22/2024). Sera were separated by centrifugation at 1500 rpm for 5 min. The serum from one unvaccinated guinea pig served as a negative control. 

### 2.4. Cross-Neutralization Assay

The sera were titrated using a viral neutralization assay, as previously described [34,35]. All hyperimmune serum stocks were normalized to a neutralizing antibody titer of 2.4 (homologous virus), using the Reed and Muench method. Cross-neutralization assays were performed in 96-well plates using Madin–Darby bovine kidney (MDBK) cells. Six serial dilutions of each virus were combined with a 1:50 dilution of the challenge serum and incubated at 37 °C for one hour. The medium was then replaced with fresh medium. The plates were incubated at 37 °C for 48 h, and cytopathic effects were recorded. Virus titers were calculated using the Reed–Muench method [36].

The neutralizing index (NI) was determined as the logarithmic difference between the virus-only and serum-virus titers. Antigenic relatedness (R) was calculated using the Archetti–Horsfall formula:

[R =√ (r1 × r2)]

where (r1) is the ratio of heterologous NI (virus 2 with antiserum 1) to homologous NI (virus 1 with its own antiserum), and (r2) is the reciprocal. r1 and r2 refer to the ratios of heterologous to homologous neutralization indices, as defined by the Archetti–Horsfall formula. R values were interpreted using Brooksby’s criteria. R values were interpreted using Brooksby’s criteria [37]: 

100%: Antigenic identity 

70–99%: Minor differences 

33–69%: Minor subtype differences 

11–32%: Major subtype differences 

0–10%: Distinct serotypes

## 3. Results

### 3.1. Genomic Diversity and Phylogenetic Analysis

Five complete BuHV-1 genomes were sequenced and deposited in GenBank (Accessions: PQ662982.1–PQ662986.1). These genomes, averaging 138,277 bp, exhibited a type D arrangement featuring a unique long (UL) and short (US) region, with the latter flanked by internal (IRs) and terminal (TRs) repeats. Annotation identified 72 open reading frames (ORFs), consistent with references BuHV-1 B6 and BoHV-5 A663 (Supplementary Table S2). The genome of Argentine strains shared nucleotide identities ranging from 96.1% to 99% with the Australian B6 strain (NC_043054.1).

**Figure 1:**
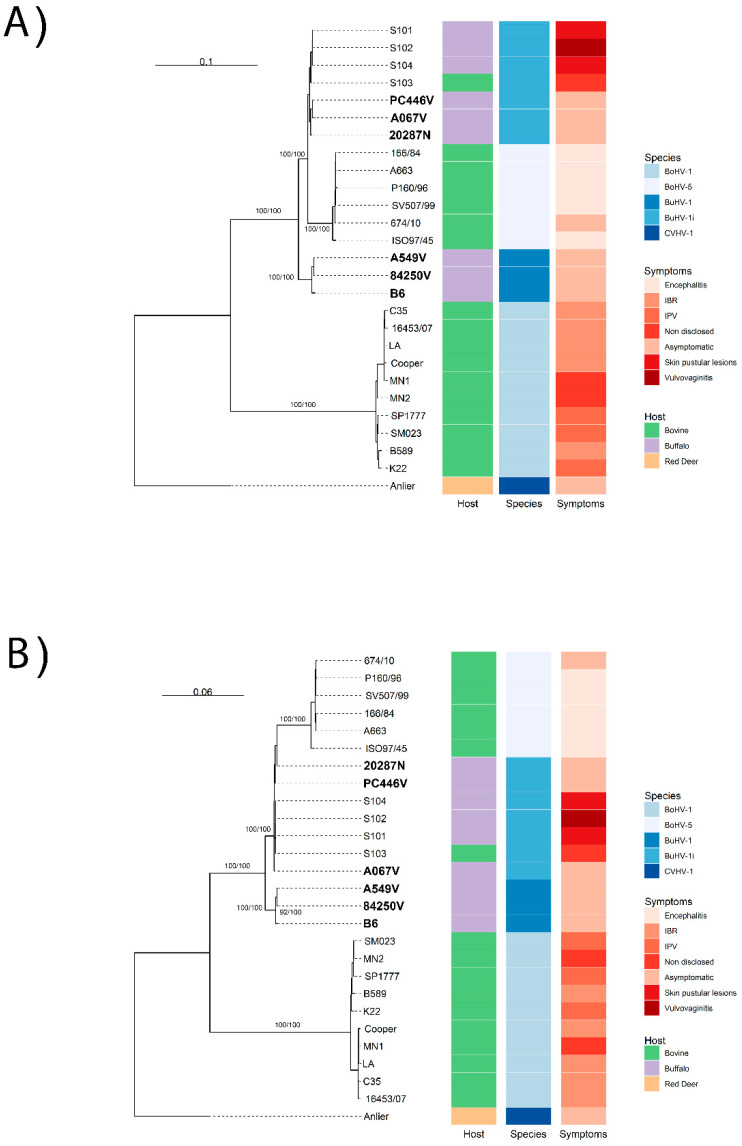
Nucleotide Phylogenetic Analyses based on the complete genome (**A**) and CDS (**B**) of BuHV-1 and BoHV-5. Maximum likelihood phylogenetic tree based on whole-genome and CDS alignment (excluding recombinant regions), with BoHV-1 and CvHV-1 as outgroups. Argentine isolates are highlighted in phylogenetic trees. The isolates, host species, and clinical signs are also displayed side-by-side for each phylogenetic tree.

**Figure 2:**
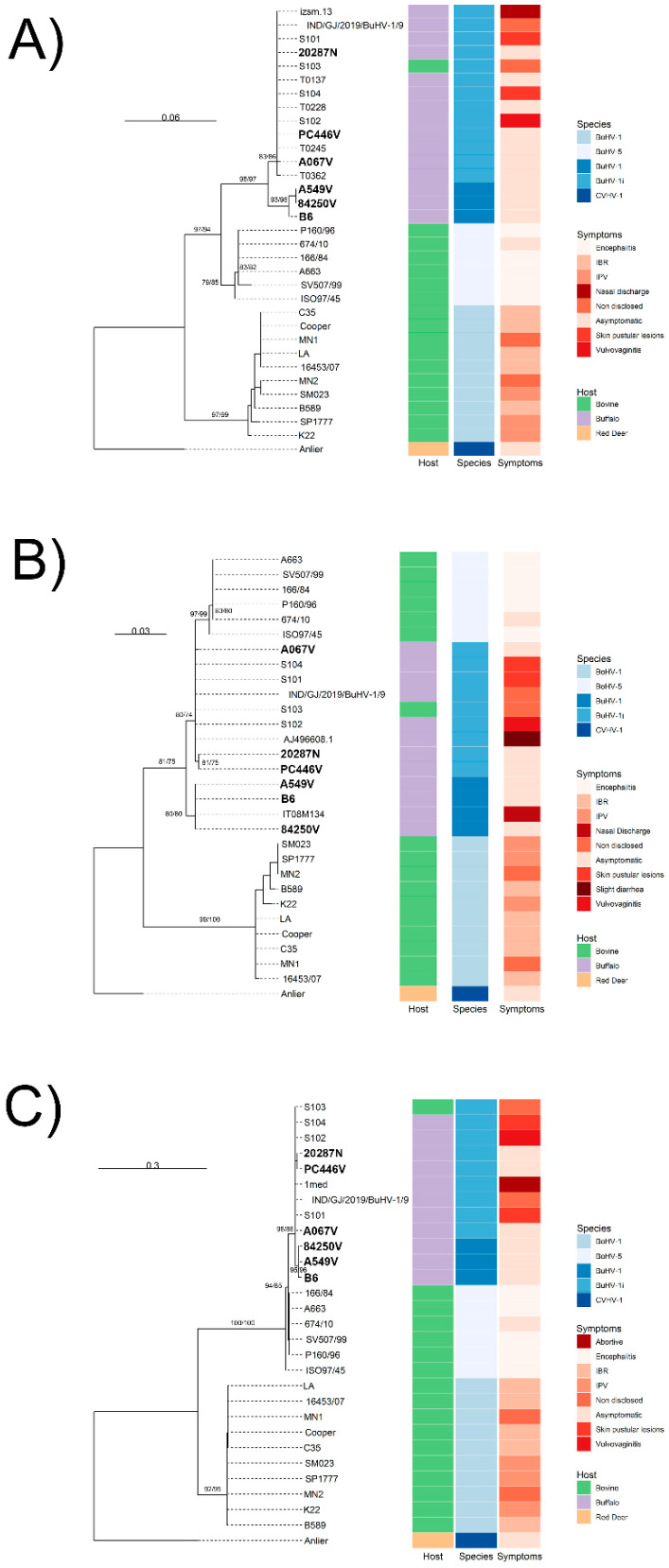
Nucleotide Phylogenetic Trees of Partial Glycoprotein Sequences. Maximum likelihood trees for (**A**) gC, (**B**) gD, and (**C**) gE sequences from BuHV-1 and BuHV-1i strains, with CvHV-1 (Anlier) as the outgroup. Argentine isolates are highlighted in phylogenetic trees.

Whole-genome and CDS phylogenetic trees (Figure 1A,B), excluding recombinant regions, revealed a BuHV-1 lineage that is distinct from BoHV-5 and BuHV-1 strains. Within BuHV-1, strains B6, A549V, and 84250V formed a clade sharing a recent common ancestor. The A067V, PC446V, 20287N, and Indian strains (S101–S104) displayed a paraphyletic basal arrangement (Figure 1A,B). These paraphyletic strains are tentatively designated as BuHV-1 indeterminate (BuHV-1i) (Figure 1). Partial gC, gD, and gE phylogenies from clinical isolates showed similar topologies, except for gC, which formed two distinct BuHV-1 subclades (Figure 2A). Only the gD fragment of the Italian strain IT08M134 clustered with B6; the other gC and gD fragments from different clinical samples aligned with BuHV-1i.

**Figure 3:**
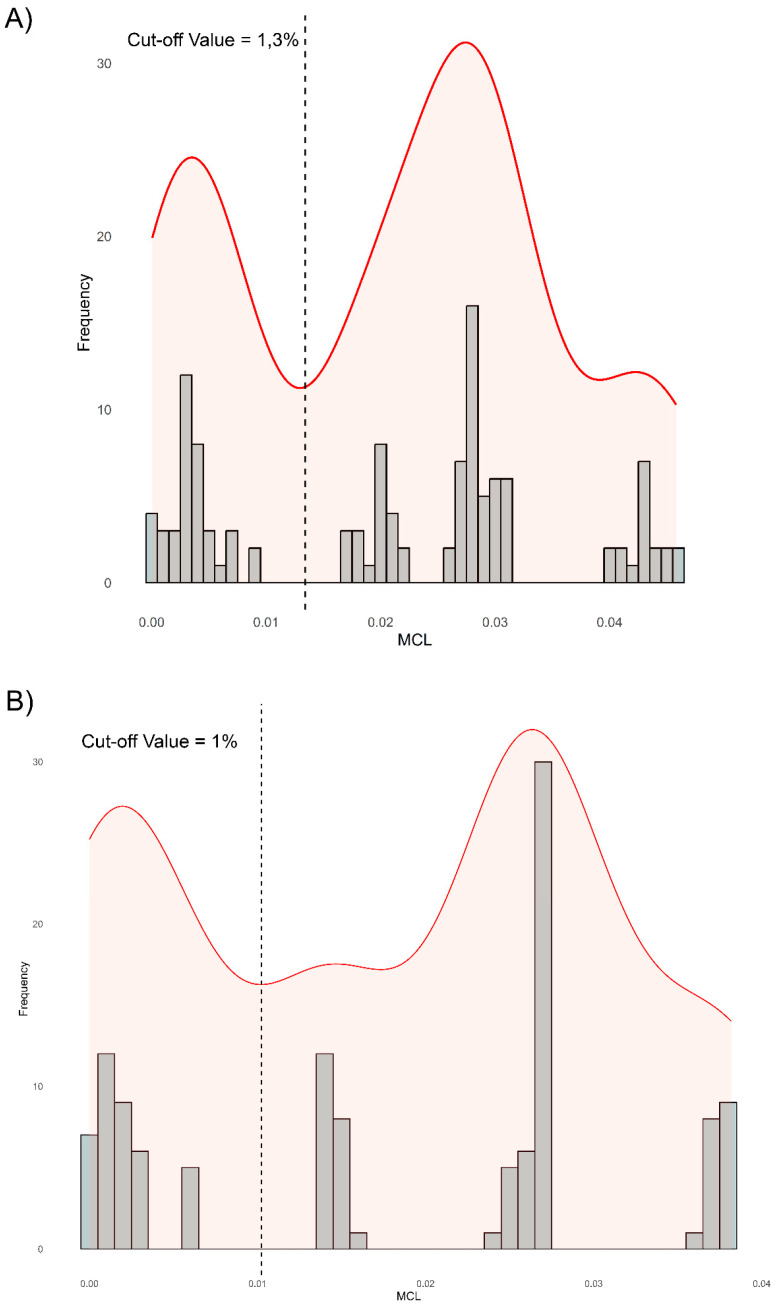
Analysis of nucleotide distances between BuHV-1 and BoHV-5 strains at the genomic level and CDS: pairwise distances in the BoHV-5, BuHV-1, and BuHV-1i alignment were calculated using Mega 11 [27], and the frequencies were plotted using the R package (https://cran.r-project.org/). A kernel density plot was also generated and combined with the distance frequencies. (**A**) Kernel density plot of whole-genome pairwise distances, with a species-level cut-off at 1.3% (vertical line). (**B**) Kernel density plot of CDS pairwise distances, with a cut-off at 1.0%.

Genomic distance thresholds were established at 1.3% for whole-genome alignments and 1.0% for CDS alignments (Figure 3A,B). The distances between BuHV-1 (B6, A549V, 84250V) and BoHV-5 exceeded these thresholds, as did the BuHV-1i strains (A067V, PC446V, 20287N, S101–S104) in relation to BuHV-1 (Table 1). These findings suggest that BuHV-1i may represent a distinct ensemble of strains from the BuHV-1 (B6, A549V, 84250V) group.

Recombination analysis identified 28 events, including 11 between BoHV-5 and BoHV-1. Among BuHV-1 strains, 84250V and 20287N exhibited recombination, with 20287N (BuHV-1i) showing nine events, primarily in the UL region (average length: 169 bp) (Supplementary Table S3). In all cases, BoHV-1 was identified as the minor parent.

### 3.2. Antigenic Characterization

Cross-neutralization assays indicated antigenic heterogeneity among BuHV-1 and BuHV-1i strains (Table 2). The R values for BuHV-1 strains ranged from 82% (minor differences) to 33% (minor subtype differences). BuHV-1i strains demonstrated a stronger antigenic relatedness to BoHV-5 (R = 70–99%) than to BoHV-1 (R = 25–26%), with major subtype differences. Strain A067V was identified as a distinct serotype (R = 0%) compared to BoHV-5 and BoHV-1. BuHV-1 strains exhibited minor subtype differences compared to BoHV-1 (R = 39–66%).

## 4. Discussion

This study presents the first complete genomic sequences of BuHV-1 strains from the Americas, thus expanding the global dataset to include ten BuHV-1 genomes. The Argentine isolates, sequenced from asymptomatic buffaloes in 2014, share 96.1–99% identity with the Australian B6 strain, highlighting both the conservation and diversity within BuHV-1. Phylogenetic analyses consistently identified a monophyletic BuHV-1 subclade (B6, A549V, 84250V) and a paraphyletic ensemble of strains referred to as BuHV-1i (A067V, PC446V, 20287N, S101–S104). The paraphyletic nature of the BuHV-1i group suggests it may represent an ancestral complex of diverging strains, potentially warranting taxonomic reclassification.

Genomic distance analyses confirmed that BuHV-1 and BoHV-5 are distinct species. BuHV-1i strains exceed species-level thresholds (1.3% of the complete genome and 1.0% of the CDS) in relation to BuHV-1 [28]. The observed divergence between BuHV-1 strains of different origins suggests that the virus may be subject to evolutionary pressures related to interspecies jumps, host adaptation, viral fitness and the different immunological status of hosts. This genetic divergence, together with recombination events between BuHV-1 and BoHV-1, particularly in the 20287N strain, supports the hypothesis that BoHV-5 could have originated from such events [13]. Identifying BoHV-1 as a minor progenitor in BuHV-1i recombination events is consistent with reports of BoHV-1/BoHV-5 co-infections in buffalo, facilitating genetic exchange [13]. Recombination between BuHV-1i and BoHV-1 in the Argentine strain 20287N raises concerns about the emergence of mosaic strains with unpredictable virulence or immunogenicity profiles, which could compromise current diagnostic tests and vaccination strategies. 

BuHV-1 infections are primarily subclinical, while BuHV-1i strains have been associated with clinical signs such as vulvovaginitis, pustular lesions, and abortions [7,10,11,12]. Strain 20287N, which was isolated from a nasal swab, exhibits mild respiratory signs in experimental infections, which may be due to its recombination with BoHV-1, a virus that is commonly recognized for its respiratory tropism [15]. The broader tissue tropism of BuHV-1i, which is present in semen, tonsils, and aborted fetuses, suggests an ecological niche differentiation that increases viral persistence in mixed agricultural systems [7,8,10,13].

BuHV-1i and BuHV-1 strains have been shown to infect cattle and goats under experimental conditions [15,21]. Additionally, the isolation of a BuHV-1i strain from naturally infected Indian cattle supports the potential for cross-species transmission under field conditions [10]. These findings underscore the risks associated with viral reservoirs in mixed ruminant systems, which complicate eradication programs and hinder diagnostic strategies due to antigenic cross-reactivity, genomic recombination, and overlapping clinical presentations [38].

Antigenic profiling revealed considerable heterogeneity, with BuHV-1i strains showing greater divergence from BoHV-1 than BuHV-1. The classification of A067V (BuHV-1i) as a distinct serotype (R = 0%) indicates unique antigenic properties, which could affect vaccine efficacy [39]. Current BoHV-1 gE-deleted vaccines offer protection against BuHV-1 but are less effective against some BuHV-1i strains, necessitating multiple doses [39,40,41,42]. This antigenic divergence underscores the need to develop targeted vaccines that specifically address BuHV-1i strains to enhance control in buffalo populations.

The results of the study highlight the need to recognize the heterogeneity among BuHV-1 strains, which requires further evaluation by the International Committee on Taxonomy of Viruses (ICTV) [43] to harmonize surveillance protocols and formal classification of circulating BuHV-1 strains [43]. The genetic and antigenic diversity of BuHV-1 strains, together with their potential for transmission and recombination between species, emphasizes the importance of having differential diagnostic tools that can distinguish between BoHV-1, BoHV-5, BuHV-1, and BuHV-1i infections in endemic regions. Additionally, priority should be given to studying and developing vaccines that protect against the full range of antigens observed in BoHV-1/5 and BuHV-1, as well as customizing prevention strategies for complex systems, such as mixed systems. As the global buffalo breeding industry grows and international trade in genetic material (semen) increases, the economic and epidemiological importance of BuHV-1 will grow. 

## 5. Conclusions

This research outlines the genetic and antigenic variation of BuHV-1, including the first genomic sequences from Argentine isolates in the Americas. A nucleotide divergence of up to 1.3% among BuHV-1 strains, combined with recombination events and antigenic diversity, reflects the virus’s complexity. The discovery of BuHV-1i as a potentially separate strain group highlights the need for better classification and specific diagnostic methods. With the increasing importance of buffalo farming and mixed agricultural systems, these results stress the importance of developing targeted surveillance, diagnostic, and vaccination strategies to mitigate the impact of BuHV-1 and its variants on livestock worldwide. 

## Figures and Tables

**Table 1:** Average nucleotide sequence distances between strains, measured across either entire genomes (top-right of the table) or coding sequences (CDS) (bottom-left of the table).

	Complete genomes MLC Distances		
	BoHV-5	BuHV-1	BuHV-1i
BoHV-5		0.0428	0.0253
BuHV-1	0.0367		0.0223
BuHV-1i	0.0262	0.0210	
	CDS MLC distances		

**Table 2:** Antigenic Relatedness (R) Values from Cross-Neutralization Assays.

Strain Comparison	R Value (%)	Antigenic Differences
BuHV-1 strains	33–82	Minor subtype differences
BuHV-1i vs. BoHV-5	70–99	Minor differences
BuHV-1i vs. BoHV-1	25–26	Major subtype differences
BuHV-1 vs. BoHV-1	39–66	Minor subtype differences
A067V vs. BoHV-5/1	0	Distinct serotype

Note: Detailed values are available in Supplementary Materials (Supplementary Table S4).

## Data Availability

Whole-genome sequences are available in GenBank under accession numbers PQ662982.1 to PQ662986.1. All other data supporting the conclusions of this article are included within the article and its Supplementary Materials.

## Supplementary Materials

The following supporting information can be downloaded at: https://www.mdpi.com/article/10.3390/v17081110/s1. Table S1: GenBank accession numbers for glycoprotein sequences used in phylogenetic analyses. Table S2: Annotation details of complete BuHV-1 genomes. Table S3: Comprehensive recombination analysis results. Table S4: Details of antigenic Relatedness (R) Values.
