# Supplementary material for: Genetic and Antigenic Diversity of Bubaline alphaherpesvirus 1
Source: Viruses. 2025 Aug 13;17(8):1110. doi: 10.3390/v17081110 (PMC12390673; doi:10.3390/v17081110)
Supplement: Supplementary file 1 [file viruses-17-01110-s001.zip › Table S1.pdf]

Table S1. Partial sequences and clinical signs of bubaline herpesvirus strains available in GenBank.

Gene	Strain name	GenBank accession number	Specie	Year	Country	Signs	Isolation source
**gC**	IZSM.13	KF679678.1	BuHV-1i	2012	Italy	Nasal mucus discharge and lesions in the nasal mucosa in four of five buffaloes experimentally infected	Nasal swab
	T0362	OR514874.1	BuHV-1i	2021	Brazil	Asymptomatic	Palatine tonsils
	T0228	OR514858.1	BuHV-1i	2021	Brazil		Palatine tonsils
	T0137	OR514857.1	BuHV-1i	2021	Brazil		Palatine tonsils
	T0245	OR514856.1	BuHV-1i	2021	Brazil		Palatine tonsils
**gD**	IT08M134	MH253681.1	BuHV-1	2008	Italy	fever, clear ocular and nasal discharge, mild cough and dyspnea. after reactivation	Vaginal swabs
	AJ496608.1	AJ496608.1	BuHV-1i	N/A	Italy	Slight diarrhea and slight serous nasal discharge after reactivation	Nasal swabs
**gE**	1med	KC202807.1	BuHV-1i	2011	Italy	Abortion (Amoroso 2013)	Aborted tissue
**gC, gD, gE**	IND/GJ/2019 /BuHV-1/9	PQ474328.1 PQ474334.1, PQ474323.1	BuHV-1i	2019	India	N/A	Semen
