# Supplementary material for: Genetic and Antigenic Diversity of Bubaline alphaherpesvirus 1
Source: Viruses. 2025 Aug 13;17(8):1110. doi: 10.3390/v17081110 (PMC12390673; doi:10.3390/v17081110)
Supplement: Supplementary file 1 [file viruses-17-01110-s001.zip › Table S2.pdf]

Table S 2: Annotation details of complete BuHV-1 genomes									
	BuHV-1		BuHV-1i						
	**84250**	**A549**	**A067**	**PC446**	**20287**	**S102**	**S101**	**S103**	**S104**
**UL55**	97,29	99,051	97,696	97,561	97561	97,696	97,696	97,696	97,696
**UL54**	98,696	99,674	98,696	98,615	98,615	98,696	98,696	98,696	98,696
**UL53**	99,9	99,9	98,996	98,695	98,695	98,896	98,896	98,896	98,896
**UL52**	99,602	99,633	98,32	98,289	98,289	98,5	98,5	98,32	98,5
**UL51**	98,9	98,9	96,3	96,3	96,5	95	95	95,9	95
**UL50**	95,284	95,284	97,24	97,412	97,24	97,365	97,365	97,365	97,365
**UL49**	97,951	97,695	95,871	96,129	95,613	96	96	96	96
**UL49,5**	100	100	98,333	98,333	98,333	98,333	98,333	98,33	98,333
**UL48**	99,789	99,789	98,192	98,401	97,636	98,261	98,261	97,636	98,261
**UL47**	99,597	99,642	97,226	97,181	97,181	97,047	97,047	97,047	97,047
**UL46**	98,699	98,699	95.434	95,042	95.073	95,168	95,168	95,035	95
**UL44**	99,295	99,295	96,627	96,697	96,697	96,89	96,890	96,767	96,89
**UL43**	99,738	99,738	98,163	98,163	97,813	98,163	98,163	98,163	98,163
**UL42**	99,757	99,838	98,229	98,148	97,987	98,148	98,148	98,148	98,148
**UL41**	99,561	99,561	98,83	99,196	99,269	99,123	99,196	99,96	99,196
**UL40**	99,895	100	99,684	99,684	99,684	99,578	99,578	99,578	99,578
**UL39**	99,665	99,707	98,585	98,627	98,627	98,627	98,627	98,627	98,627
**UL38**	98,745	98,466	97,978	98,047	98,047	98,047	98,047	98,047	98,047
**UL37**	99,462	99,62	98,293	99,052	98,831	99,083	99,083	99,083	99,083
**UL36**	97,134	98,796	96,251	96,119	96,133	97,395	97.395	97	97,395
**UL35**	99,471	100	99,735	99,735	91,063	99,471	99,471	90,821	99,471
**UL34**	97,143	99,879	97,024	97,024	96,31	97,024	97,024	97,024	97,024
**UL33**	100	100	99,399	99,399	99,399	99,399	99,399	99,399	99,399
**UL32**	99,779	99,779	98,075	99,227	99,227	99,118	99,118	99,118	99,118
**UL31**	99,91	99,91	97,222	97,67	97,67	97,67	97,67	97,67	97,67
**UL30**	99,087	99,007	98,062	98,089	98,089	97,82	97,82	97,82	97,82
**UL29**	99,532	99,807	99,422	99,312	99,312	99,257	99,257	99,257	99,257
**UL28**	99,53	99,615	99,487	99,487	99,847	99,444	99,444	99,444	99,444
**UL27**	99,436	99,471	98,523	98,523	97,066	99,121	99,121	99,121	99,121
**UL26**	99,605	99,577	97,2	97,468	97,365	97,538	97,538	97,538	97,538
**UL25**	99,23	99,614	99,12	99,227	99	99,175	99,175	99,175	99,175
**UL24**	98,13	99,377	97,257	97,382	97,382	97,506	97,631	97,506	97,506
**UL23**	96,919	99,627	96,732	96,732	96,732	96,732	96,732	96,732	96,732
**UL22**	99,442	99,642	98,535	98,298	97,557	98,496	98,574	98,496	98,496
**UL21**	99,943	99,943	96,156	96,156	96,585	98,07	98,07	95,947	98,07
**UL20**	99,573	100	97,436	97,436	97,436	97,436	97,436	97,436	97,436
**UL19**	99,833	99,857	99,142	99,142	99,142	99,165	99,022	99,165	99,165
**UL18**	99,579	99,579	99,369	99,264	99,264	99,369	99,369	99,369	99,369
**UL15-17**	99,909	99,909	99,453	99,407	99,407	99,453	99,453	99,453	99,453
**UL17**	99,599	99,85	97,822	97,921	98,064	97,921	97,921	97,921	97,921
**UL16**	99,419	99,419	99,516	99,516	99,516	99,225	99,225	99,225	99,225
**UL14**	99,399	99,399	97,536	97,837	96,984	98,07	98,07	95,793	98,07

**UL13**	99,908	99,908	98,801	98,708	98,708	98,893	98,893	98,893	98,893
**UL12**	99,459	99,459	97,513	97,513	96,761	96,909	96,909	96,909	96,909
**UL11**	100	100	92,578	92,188	92,188	94,606	94,606	92,578	96,68
**UL10**	97,937	99,206	98,095	98,175	98,175	98,016	98,016	98,016	98,016
**UL9**	99,434	99,838	99,071	99,111	99,111	99,111	99,111	99,111	99,111
**UL8**	99,237	100	98,16	98,205	98,205	98,205	98,205	98,205	98,205
**UL7**	99,89	99,89	98,675	98,675	98,675	98,675	98,675	98,675	98,675
**UL6**	99,769	99,494	97,726	98,579	98,073	98,487	98,579	96,107	98,487
**UL5**	99,28	99,519	98,254	98,214	99,197	99,077	99,077	99,116	99,077
**UL4**	100	100	98,765	98,589	98,589	98,765	98,765	98,765	98,765
**UL3,5**	91,019	91,262	99,202	99,202	99,202	99,202	99,202	99,202	99,202
**UL3**	99,692	99,692	93,684	93,835	93,835	96	96	93,835	96
**UL2**	98,765	98,765	96,656	94,444	94,444	96,656	96,656	96,656	96,656
**UL1**	99,79	99,79	97,275	97,484	97,484	97,484	97,484	97,484	97,484
**BICP0**	98,492	98,492	94,563	94,5	94,556	95,377	95,377	94,25	95,377
**BICP4**	97,492	97,601	89,57	89,589	93,149	95,332	95,36	94,025	95,356
**BICP22**	98,313	98,758	90	91,731	89,123	87,652	86,99	86,99	86,99
**US 1,6**	100	100	95,699	95,699	95,699	95,43	95,43	95,43	95,43
**US2**	99,266	99,559	97,504	97,21	97,21	97,21	97,21	97,21	97,21
**US3**	97,993	99,274	95,28	95,352	93,352	95,062	95,062	95,062	95,062
**US4**	99,321	99,321	95,441	95,871	95,724	95,366	95,366	95,366	95,366
**US6**	100	100	98,102	98,102	98,102	98,102	98,102	98,102	98,102
**US7**	99,312	99,398	97,332	97,418	97,418	97,246	97,246	97,246	97,246
**US8**	99,769	99,769	97,19	96,961	96,951	97,018	97,353	97,018	97,018
**US9**	99,449	99,449	96,727	95,324	95,818	96,545	96,545	96,545	96,545
**BICP22**	99,383	99,814	89,505	90,832	87,849	85,766	85,887	85,766	85,766
**BICP4**	97,144	95,015	90,435	91,569	91,766	94,22	94,137	92,723	94,11
