# Supplementary material for: Genetic and Antigenic Diversity of Bubaline alphaherpesvirus 1
Source: Viruses. 2025 Aug 13;17(8):1110. doi: 10.3390/v17081110 (PMC12390673; doi:10.3390/v17081110)
Supplement: Supplementary file 1 [file viruses-17-01110-s001.zip › Table S3.pdf]

**Table S4.** Interspecific recombination between Bubaline alphaherpesviruses				
**Recombinant strain**	**Major parental**	**Minor parental**	**Breakpoint***	**Gene/s**
**A663, 166/84 674/10**	BoHV-5	BoHV-1	56964-59196	UL27**
**A663, 166/84, P160/96, ISO97/45**	BoHV-5	BoHV-1	85636-88710	UL11-UL9***
**A663, 166/84, 674/10, ISO97/45, SV507/99, P160/96, Bhilwara**	BuHV-1	BoHV-1	8933-9222	UL50
			11269-15643	UL48-UL46
			17903-18132	UL44
			36410-36919	UL36
			48570-48884	UL30
			71963-72526	UL19
			94633-97492	UL6-5****
			107850-108475	ICP4
			133341-133966	ICP4
**84250V (BuHV-1)**	BuHV-1	BuHV-1i	126-2829	UL55 – UL54
			27874-30239	UL38 – UL37
			38933-42288	UL36-UL34
			53277-55335	UL29 – UL28
			62070-64682	UL25 – UL24
			66109-66702	UL22
			78022-79192	UL17
			85685- 90544	UL10 – UL8
**20287N (BuHV-1i)**	BuHV-1i	BoHV-1	9072-9169	UL50
			12199-12408	UL48
			14888-14986	UL47
			58058-58138	UL27
			82790-83070	UL14
			84386-84498	UL12
			85252-85399	UL12
			108093-108341	ICP4
			134617-134865	ICP4

*Breakpoint locations are based on the first genome mentioned for each event.
** Previously reported in Maidana et al., 2017
*** Previously reported in Romera et al., 2022. Partial recombination event (shorter length) in ISO97/45 and P160/96.
****Partial recombination event (shorter length) in ISO97/45.
