# Supplementary material for: Genetic and Antigenic Diversity of Bubaline alphaherpesvirus 1
Source: Viruses. 2025 Aug 13;17(8):1110. doi: 10.3390/v17081110 (PMC12390673; doi:10.3390/v17081110)
Supplement: Supplementary file 1 [file viruses-17-01110-s001.zip › Table S4.pdf]

Table S4. Antigenic relatedness (R)* of BuHV-1, BuHV-1i, BoHV-1 and BoHV-5 strains (%)

	Viruses						
	BoHV-1	BoHV-5	BuHV-1			BuHV-1i	
**Antiserum**	**LA**	**2010**	**B6**	**A549V**	**84250V**	**PC446V**	**A067V**
**2010**	33	…					
**B6**	66	91	…				
**A549V**	39	89	63	…			
**84250V**	72	100	100	100	…		
**PC446V**	26	44	57	63	82	…	
**A067**	0	0	63	93	54	63	…
**20287N**	25	50	33	55	63	92	49

*R = 100% Antigenic identity between two viruses
R = 70-99% Minimal or no difference between two viruses
R = 33-69% Minor subtype differences between two viruses
R = 11-32% Major subtype differences between two viruses
R = 0-10% Different serotypes
